# Incidence of adverse drug events in patients hospitalized in the medical wards of a teaching referral hospital in Ethiopia: a prospective observational study

**DOI:** 10.1186/s40360-022-00570-w

**Published:** 2022-05-17

**Authors:** Teketel Alemu Ersulo, Mengist Awoke Yizengaw, Behailu Terefe Tesfaye

**Affiliations:** 1 Hadiya Zone, West Badewacho Woreda Health Office, Addis Ababa, Ethiopia; 2 School of Pharmacy, Clinical Pharmacy Unit, Jimma University, Institute of Health, P.O.B: 378, Jimma, Ethiopia; 3 Jimma University Medical Center, Institute of Health, Jimma, Ethiopia

**Keywords:** Adults, Medication, Undesirable events, Predictors

## Abstract

**Background:**

Adverse drug events (ADEs) are an important public health problem with considerable clinical and economic costs. However there are limited studies of ADE incidence in adult inpatients in low-income countries, particularly in Ethiopia. Hence, this study aimed to assess the incidence of adverse drug events and associated factors in patients hospitalized in the medical wards of Wolaita Sodo University teaching referral hospital (WSUTRH).

**Methods:**

A prospective observational study was conducted involving 240 patients admitted to the medical wards of WSUTRH. A checklist was used for data collection, while standard tools were employed for assessing the probability and characterization of ADEs. A multifaceted approach involving daily chart review, patient interview, attendance at ward rounds and/or meetings, and staff reports were employed to collect the data. To identify factors independently associated with ADEs, logistic regression analysis was conducted using Stata version 15.

**Results:**

Patients were followed from ward admission to discharge, accounting for 2200 patient-days of hospital stay. Overall, 976 medications were ordered during the hospital stay. Sixty-four ADEs were identified with an incidence of approximately 27 per 100 admissions and 29 per 1000 patient days. Of the total ADEs, 59% were preventable. Regarding the severity, 2% of the ADEs were severe, while 54% were moderate. The risk of ADEs increased with longer hospital stay (LOHS) (*p* = 0.021), in patients with blood and immune disease diagnosis (*p* = 0.001), use of cardiovascular medicines (*p* = 0.028), and an increase in the number of medications prescribed (*p* = 0.021).

**Conclusions:**

In this study, ADEs were identified in about one-quarter of the participants. Longer hospital stays, blood and immune diseases, cardiovascular medicines use, and multiple medication use had increased the likelihood of ADE occurrences. The majority of the ADEs were preventable, indicating the existence of a window of opportunity to ensure patient safety.

**Supplementary Information:**

The online version contains supplementary material available at 10.1186/s40360-022-00570-w.

## Introduction

The evolution of clinical therapeutics has positively affected public health, but these benefits have also been accompanied by increased risks of medical harm [1, 2]. Adverse drug event (ADE) is the most common cause of medical harm. It is defined as an injury resulting from the use of medication [3], which can be preventable or non-preventable [4]. It is estimated that about half of ADE incidences are preventable [2, 5–8].

There are various strategies to detect ADEs in hospital settings. The most common methods involve spontaneous reporting systems, patient interviews and chart reviews, trigger tools, and computerized monitoring systems [9, 10]. The traditional, spontaneous reporting system is presumed ineffective because of factors such as under-reporting and lack of essential data for identification of the causality of suspected drugs [10]. There is no single standard method to employ, thus the use of multiple strategies to maximize the detection of ADE incidence is recommended [11].

Globally, ADEs are among the major public health concerns with variable incidence reports across the studies. For instance, a study from Japan reported 29.2% [12], a study from Saudi-Arabia revealed 8.5% [13], in Africa, studies from Uganda recorded 25% [14], while a study from Ethiopia reported 36.4% [15] ADE incidences. These variable reports are attributed to factors such as differences in the strategies employed for detecting ADEs [9, 10], the specific study definition of ADEs, and others. Adverse drug events often lead to hospital admission [16–25], prolongation of hospital stay [5, 6, 26–29] and mortality [26], increasing the healthcare expenditures [20, 26, 30–32]. Healthcare professionals are also indirectly affected by the ADEs because of the loss of public’ confidence in the health service [33]. Thus, provided that more than half of ADE incidences are preventable [2, 5–8], it is highly imperative to identify ADE risk factors and prevent their incidences. Various studies had reported sex, age, length of hospital stay, comorbidity, specific drug class, medication error, and the number of medications as the major risk factors for ADEs incidences [6, 14, 34–37].

There are limited studies on ADEs in the Ethiopian hospital settings. In a cross-sectional study on adverse drug reaction-related hospitalization from Jimma University medical centre, adverse drug reactions accounted for 10.3% of hospital admissions [38]. Another study from the same setting found that above quarter of patients (26.6%) admitted to the hospital experienced ADEs during their hospital stay [15]. Because of the scarce availability of such studies in Ethiopia, this study was proposed and conducted to assess the incidences and determinants of ADE among patients hospitalized in the medical wards.

## Material and methods

### Study area and period

The study was conducted from February 2021 to July 2021, in the medical wards of WSUTRH, in Sodo town, Southwest Ethiopia. Sodo town is 330 km away from the national capital, Addis Ababa. The hospital has 1019 staff; 418 professionals, and 601 support staff. The hospital provides health services for about 12,944 inpatients and 109,091 outpatients per year. There are two medical units under the medical ward, i.e., the male and the female ward. The medical ward had 3 internists, 3 general practitioners, 10 nurses, and 2 clinical pharmacists. It has 3 rooms and 49 beds for both male and female adult inpatient services.

### Study design

A prospective observational study design was employed.

### Source and study population

#### Source population

All adult inpatients from the medical wards of WSUTRH.

#### Study population

All adult patients admitted to the medical wards of WSUTRH during the data collection period who fulfilled the inclusion criteria.

#### Eligibility criteria

All patients aged ≥18 years old were included in the study, while patients who declined to take part in the study, with hospital stay < 24 hrs, and who lost follow up were excluded.

### Sample size and sampling procedure

Sample size (n) was calculated using a single population proportion formula considering the proportion of ADE occurrence (P) of 0.36 [15]; level of confidence (Z = 1.96) of 95%; the size of patients admitted in the previous 6 months before this study (N) =640; and margin of error (W) of 5%. The final sample size was 240.

### Study variables

Incidence of ADE was a dependent variable, while the independent variables were: sociodemographic and behavioural variables **[**Age**,** sex, residence**,** marital status**,** educational status**,** occupation**,** alcohol use**,** cigarette smoke**,** and traditional medicine use history], clinical and related factors [history of hospitalization in the previous 3 months, past medical history, current diagnosis, Charlson’s comorbidity index score, and LOHS], medications and related factors [past medication history, ADE history, number of medications, and class of medication used].

### Data collection tools, procedures, and case detection

The data collection checklist employed in the present study was developed after reviewing relevant literature. The checklist comprises sociodemographic, behavioural, medication, and clinical-related variables. Initially, the data collection checklist was designed in English, then some parts of the tool (the ones that were used directly to collect information from patients or attendants, like socio-demographic, informed consent, and patient information sheet) were translated to two locally dominant languages (Wolaitigna and Amharic) and back to English.

Two clinical pharmacists were employed as data collectors, while one internal medicine resident was employed as a supervisor. The data collectors enrolled and interviewed eligible participants and reviewed medical charts daily for all admissions. They strictly followed changes in medication experiences and abnormal laboratory values to identify incidences of ADEs. The medical ward staff and patients were briefed on the objectives of the study and requested cooperation to report in case of any event occurrence (self-report). Besides this, a standard trigger tool [39], which involves drugs or clues that have links to potential ADEs because either they are antidotes or given to reverse the action of a drug responsible for ADE, was used to increase the ADEs detection rate. The responsible physician was contacted in case of medication management changes to clarify the changes made. Also, the clinical pharmacists attended clinical rounds and visited the wards daily to solicit any alerts for ADE. Patients were interviewed using a questionnaire that contains sociodemographic and behavioural variables, medical and medication history. The clinical pharmacist forwarded any suspected ADE cases for further evaluation to a multidisciplinary team composed of physicians (internists, general practitioners), ward pharmacists, and clinical nurses. When ADE was detected, the data collectors recorded the event in the ADE documentation format.

### Methods for classifying diagnosis, medications prescribed, and medication-related events

In the present study, diagnosed diseases are classified according to the international classification of diseases, tenth edition (ICD-10) [39], and scored using the Charlson comorbidity index (CCI) score [40]. The prescribed medications are categorized in line with anatomical, therapeutic, and chemical classification. During patient follow-up in the ward, in case of suspicion on the medication safety events, they were further evaluated and classified.

### Adverse drug event case evaluation

In this study, the term ADE is contextualized as an injury resulting from the use of medication at doses used for prophylaxis, diagnosis or therapy [41]. We employed standard tools to assess the causality, categorize the severity and determine the preventability of the ADEs. The causality of all suspected ADEs was assessed using the modified Naranjo Causality Scale (MONARCS) [42]. This validated tool scores the likelihood that an event is drug-related. The algorithm comprises questions that evaluate factors like the temporal association of drug administration and ADE occurrence, response to de-challenge or re-challenge, alternative explanations for the event, any objective evidence, and previous drug exposure. They are answered as either yes, no, or do not know. Different point values (− 1, 0, + 1 or + 2) were assigned to each answer that yields the following associations between total score and causal relationship: [1] 1 through 4 points equals possible [2]; 5 through 8 points equals probable; and [3] 9 or more points equals definite.

The severity of the ADEs was categorized based on the modified Hartwig Severity Assessment Scale [43]. Based on this scale, the severity of ADEs was classified as mild (level 1 and 2), moderate (level 3 and 4), or severe (level 6), depending on factors like requirements for change in medication, increase in in-hospital stay and led to permanent injury. The Preventability of the ADEs was determined using the explicit criteria developed by Schumock and Thornton [42]. The tool has three sections, namely definitely preventable, probably preventable, and non-preventable. Section A comprises five questions, while section B has four questions. All the answers were categorized as yes or no. Adverse drug events were definitely preventable if the answer is yes to one or more questions in section A. If the answers were all negative, the assessors will proceed to section B. Adverse drug event is probably preventable if the answer is yes to one or more questions in section B. If answers were all negative, section C proceeded. In Section C, the ADEs were non-preventable. The categorization of events (causality, severity, and preventability) was further strengthened by involving responsible clinicians. The reviewers reached a consensus through discussion in case of discordant classification.

### Data processing and analysis

Epi-Data version 4.6.0.2 and Stata version 15 were employed for data entry and analysis, respectively. Frequency and percentages were used to present categorical variables, whereas continuous variables were described using mean ± Standard deviation (SD). Outcomes were reported in terms of the institute for health care improvement (IHI) ADE reporting metrics: ADE incidence per 100 admissions, per 1000 person-days [39], per 100 medications ordered, and proportions. The incidence of ADEs per 100 admissions and 100 medications ordered was calculated by dividing the total number of ADEs identified by the total number of admissions and a total number of medications ordered respectively and multiplied by 100. Similarly, the incidence of ADEs per 1000 person-days was calculated as the total number of ADEs identified divided by the total number of patient days multiplied by 1000.

Before running regression analysis, cell adequacy was checked for each categorical variable. Variables were tested for multicollinearity by collinearity diagnostics. Then, bivariate logistic regression analysis was carried out to recruit candidate variables for multivariate logistic regression analysis. Thus, variables with a *p*-value < 0.25 were inserted into a multivariate logistic regression to identify statistically significant predictors of ADE occurrence. Crude and adjusted Odds ratio, including p-value and 95% confidence interval, was reported. A *p*-value < 0.05 was considered statistically significant.

### Quality assurance

To ensure the quality of the data, data collectors were trained on the objectives of the study, the data collection checklist, and how to assess and record ADEs before starting the work. During the actual data collection, they were evaluated & supported when demand ensues, especially on how and which data to collect from the patient chart. Supervision and daily checkup of filled data collection forms were done. The ward staff were informed of the aim of the study to facilitate the report habit of ADE incidences. Additionally, frequent consultations of the medical ward staff was done to stimulate further medication safety event/incident report verbally or to use the designed reporting format to maximize data yield. The quality of the data was assured by doing a pretest on 5% of the actual sample size before the actual data collection to check the consistency and validity of the data collection format.

## Results

### Overview

**Fig. 1:**
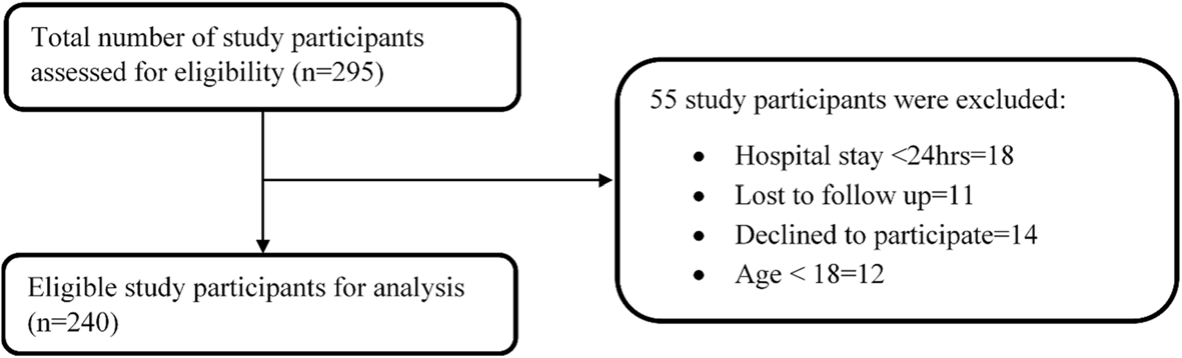
Summary of patients approached and included in the study, February 2021 to July 2021

Overall, 295 patients were approached during the study period. Of these, 55 patients were rationally excluded and 240 patients were included in the study (Fig. 1).

### Socio-demographic and behavioural characteristics of the participants

The overall mean age of participants in this study was 44 (±18) years. Most participants (52.5%) were female. Regarding residence, both rural and urban residents accounted for an equal proportion (50%). Relatively, most participants in this study attended primary school (40.8%) and merchants (27.5%) (Table 1).

**Table 1:** Sociodemographic and behavioural characteristics of study participants at WSUTRH, February 2021 to July 2021

Variable		Frequency	%
Sex	Male	114	47.5
	Female	126	52.5
Age, year		44 (±18)	
Residence	Urban	120	50.0
	Rural	120	50.0
Marital status	Married	191	79.6
	Single	47	19.6
	Divorced	2	0.83
Educational status	No formal education	51	21.2
	Primary	98	40.8
	Secondary	78	32.5
	Tertiary	13	5.4
Occupation	Student	41	17.1
	Gov’t employee	58	24.2
	Merchant	66	27.5
	Self-employed	15	6.2
	Farmer	26	10.8
	Unemployed	24	10.0
	NGO	7	2.9
	Retired	3	1.2
Alcohol use history		7	2.9
Cigarette smoke history		7	2.9
Traditional medicine use history		8	3.3

*NGO* Non-Governmental Organization

### Clinical and related factors of the study participants

Above two-thirds of the participants (67.5%) had a past hospitalization history. According to the ICD-10 disease category, diseases of the digestive system (19.2%), infectious and parasitic diseases (17.5%), and diseases of the respiratory system (14.6%) were the three most common diseases recorded. After admission to the wards, diseases of the circulatory system (42.1%), diseases of the respiratory system (38.3%), diseases of the blood and immune mechanism (30%), and diseases of the digestive system (30%) were the most common. The mean Charlson’s comorbidity index score was 2.0 (±2.13). The participants’ average length of hospital stay (LOHS) was 9 (±6.6) days, and about 2.1% of the patients had a clinical outcome of death (Table 2).

**Table 2:** Clinical and related factors of the study participants at WSUTRH, February 2021 to July 2021

Variables		Frequency	%
History of hospitalization in the previous 3 months		162	67.5
Past medical history based on ICD-10 code			
K00-K95	Diseases of the digestive system	46	19.2
A00-B99	Infectious and parasitic diseases	42	17.5
J00-J99	Diseases of the respiratory system	35	14.6
I00-I99	Diseases of the circulatory system	33	13.7
E00-E89	Endocrine, nutritional, and metabolic diseases	3	1.2
G00-G99	Diseases of the nervous system	3	1.2
C00-D49	Neoplasms	2	0.8
Current diagnosis based on ICD-10 code			
I00-I99	Diseases of the circulatory system	101	42.1
J00-J99	Diseases of the respiratory system	92	38.3
D50-D89	Diseases of the blood and immune mechanism	72	30.0
K00-K95	Diseases of the digestive system	72	30.0
A00-B99	Infectious and parasitic diseases	61	25.4
N00-N99	Diseases of the genitourinary system	45	18.7
E00-E89	Endocrine, nutritional, and metabolic diseases	27	11.2
G00-G99	Diseases of the nervous system	22	9.2
L00-L99	Diseases of the skin and subcutaneous tissue	6	2.5
C00-D49	Neoplasms	5	2.1
CCI score		2.0 ± 2.1	
LOHS		9 ± 6.6	
Number of deaths		5	2.1

*CCI* Charlson comorbidity index, *ICD-10* International Classification of Diseases 10th version, *LOHS* Length of hospital stay, *SD* Standard deviation, *WSUTRH* Wolaita Sodo University teaching referral hospital

### Medications and related factors of study participants

Of the included patients, 46.7% of them had a history of medication use during the 3 months before the study period. Of them, most patients were taking antibiotics (25%), gastrointestinal medicines (14.2%), cardiovascular medicines (12.9%), and antimalarial (12.5%). During admission, above one quarter (27%) of the patients were taking medication. During the entire hospital stay (current medications), 976 medications have been ordered and, on average, each patient has been prescribed 4 (±1.9) medications. Of these, antibiotics (65%) were the leading class, followed by gastrointestinal medicines (33.3%) (Table 3).

**Table 3:** Medication and related factors of study participants at WSUTRH, February 2021 to July 2021

Variables	Frequency	%
History of medication in the past 3 months	112	46.7
Taking medications during admission	65	27.1
Had history of adverse drug event(s)	11	4.6
Past medication history		
Antibiotics	60	25
Gastrointestinal medicines	34	14.2
Cardiovascular medicines	31	12.9
Antimalarials	30	12.5
Analgesics	8	3.3
Antituberculosis	7	2.9
Antiasthmatic	6	2.5
Antianaemic agents	6	2.5
Antiepileptic drugs	3	1.2
Antipsychotic	3	1.2
Antithyroid agents	3	1.2
Anticoagulants	2	0.8
Current medications		
Antibiotics	156	65
Gastrointestinal medicines	80	33.3
Antimalarials	53	22.1
Vitamins and anti-anaemic agents	44	18.3
Cardiovascular medicines	23	9.6
Antidiabetic agents	20	8.3
Steroids	16	6.7
Anticoagulants	14	5.8
Antiepileptic drugs	14	5.8
Analgesics	10	4.2
Antipsychotic agents	7	2.9
Antiplatelets	6	2.5
Anti-dyslipidemia agents	8	3.3
Antituberculosis agents	5	2.1
Anti-asthma agents	3	1.2
Antiviral agents	2	0.8
Number of medications	4.1 ± 1.9	

### Adverse drug events and related factors

#### Incidence of ADEs

The study participants accounted for 2200 patient days, and 64 ADEs were identified in 58 (24.2%) patients. The incidence of ADEs was approximately 27 (95% CI, 21.03–32.30) per 100 admissions, 29 per 1000 patient days, and 6 per 100 medication orders.

#### Causality, severity, and preventability of ADEs

Using the Naranjo causality assessment algorithm for ADE, 15.5% of ADEs were definite, 68.9% ADEs were probable, and 15.5% of ADEs were possible. According to the modified Hartwig ADE severity assessment scale, 43.7% ADEs were mild, 54.7% ADEs were moderate, and 1.6% ADEs were severe (Table 4).

**Table 4:** Severity of ADEs based on the modified Hartwig ADEs Severity Assessment Scale, WSUTRH, February 2021 to July 2021

Level^a^	Description	Frequency	%
1	An ADE occurred but required no change in treatment with the suspected drug	15	23.4
2	The ADE required that treatment with the suspected drug be held, discontinued, or otherwise changed. No antidote or other treatment requirement was required. No increase in hospital stays.	13	20.3
3	The ADE required that treatment with the suspected drug be held, discontinued, or otherwise changed AND/OR an antidote or another treatment was required. No increase in hospital stays.	23	35.9
4	Any Level 3 ADE which increases the length of stay by at least 1 day.	12	18.7
5	Any level 4 ADE which requires intensive medical care.	0	0
6	The ADE caused permanent harm to the patient	1	1.6
7	The ADE which led to the death of the patient	0	0
Total		64	100

^a^1&2 = mild, 3&4 = moderate, 5–7 = severe

*ADES* Adverse drug events, *WSUTRH* Wolaita Sodo University teaching referral hospital

Assessing ADEs preventability using the modified Schumock and Thornton preventability criteria, 28.1% ADEs were definitely preventable, 31.3% ADEs were probably preventable, and 40.6% of the ADEs were non-preventable

#### System organ class affected by ADEs

The Gastrointestinal (27%) and central nervous (23%) systems were the most frequently affected organ systems by the ADEs, followed by the endocrine and metabolic and cardiovascular systems (each 10.9%) (Table 5).

**Table 5:** Classification of incident ADEs by organ system, WSUTRH, February 2021 to July 2021

Organ system	Incidence	Adverse drug events	Medications
Gastrointestinal	17 (26.7)	Dyspepsia 3 (4.7)	Diclofenac (2), atorvastatin (1)
		Upper GI bleeding 2 (3.1)	Aspirin (1), diclofenac (1)
		Diarrhoea 3 (4.7)	Clarithromycin (1), Omeprazole (2)
		Vomiting 5 (7.8)	Digoxin (1) iron sulfate (4)
		Loss of appetite 2 (3.1)	Aspirin (1), atorvastatin (1)
		Constipation 2 (3.1)	Haloperidone (1) tramadol (1)
Central nervous system	15 (23.4)	Loss of consciousness 1 (1.6)	Furosemide (1)
		Sedation 2 (3.1)	Diazepam (2)
		Dizziness 2 (3.1)	Furosemide (2)
		Seizure 3 (4.7)	Insulin (1), tramadol (2)
		Hallucination 3 (4.7)	Phenytoin (1), Phenobarbitone (2)
		Headache 4 (6.2)	Nifedipine (2), enalapril (2)
Endocrine and metabolic	7 (10.9)	Hyperkalemia 1 (1.6)	Spironolactone (1)
		Hyperglycemia 1 (1.6)	Dexamethasone (1)
		Hypocalcemia 2 (3.1)	phenytoin (1) furosemide (1)
		Hypokalemia 2 (3.1)	Digoxin (1), furosemide (1)
		Hypoglycemia 1 (1.6)	Insulin (1)
Cardiovascular system	7 (10.9)	Hypotension 2 (3.1)	Furosemide (2)
		Peripheral oedema 1 (1.6)	Nifedipine (1)
		Bradycardia 3 (4.7)	Propranolol (2) digoxin (1)
		Shock 1 (1.6)	Propranolol (1)
Neuromuscular and skeletal	5 (7.8)	Muscle stiffness 3 (4.7)	Chlorpromazine (3)
		myalgia 2 (3.1)	Atorvastatin (2)
Dermatologic	5 (7.8)	Skin rash 3 (4.7)	amoxicillin/ clavulanate (Augmentin) (1), cotrimoxazole (2)
		Injection site phlebitis 2 (3.1)	Erythromycin (1), vancomycin (1)
Respiratory	4 (6.2)	Cough 2 (3.1)	Enalapril (2)
		Loss of hearing 1 (1.6)	Gentamycin (1)
		respiratory depression 1 (1.6)	Phenobarbitone (1)
Immune system	2 (3.1)	Allergy 2 (3.1)	Ibuprofen (1), aspirin (1)
Hematologic	2 (3.1)	Nose bleeding 1 (1.6)	Enoxaparin (1)
		Anemia 1 (1.6)	Artesunate (1)
Total	64 (100)		

*ADE* Adverse drug event

#### Medications and related factors

**Fig. 2:**
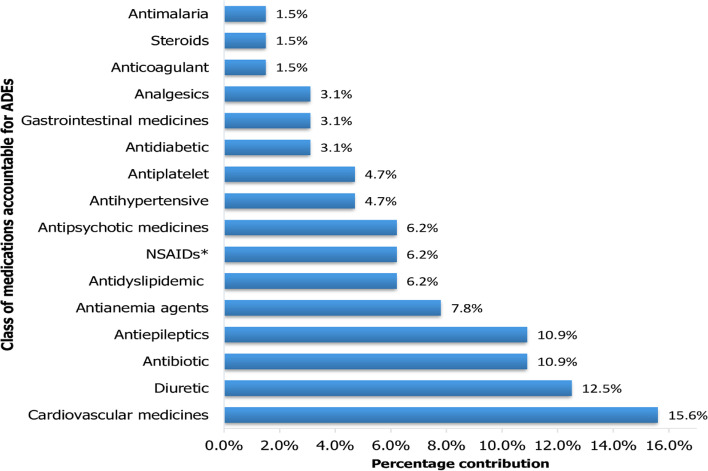
Class of medications accountable for ADEs, WSUTRH, February 2021 to July 2021. *NSAIDs, Non-steroidal anti-inflammatory drugs

In terms of class, most ADEs were caused by cardiovascular medicines, diuretics, antiepileptics, antibiotics, and anti-anaemic agents. Diuretics, cardiovascular, antiepileptic, and antibiotic medicines accounted for about 50% of ADEs (Fig. 2).

### Factors associated with the occurrence of ADEs

In the bivariate logistic regression analysis, nineteen variables had a *p*-value < 0.25 and were recruited for multivariate logistic regression. Finally, four variables were independently associated with ADE incidence. Blood and immune disease diagnosis (AOR = 3.925, 95% CI: 1.709–9.013, *p* = **0.001**), LOHS (AOR = 1.066, 95% CI: 1.009–1.125, *p* = **0.021**), cardiovascular medicines prescription during the study period (AOR = 3.368, 95% CI: 1.137–9.979, *p* = **0.028**), and the number of medications prescribed (AOR = 1.310, 95% CI: 1.041–1.647, *p* = **0.021**) increased the risk of ADE incidences (Table 6).

**Table 6:** Patient-related factors associated with ADE occurrence in the medical ward of WSUTRH, February 2021 to July 2021

Disease and drug related variables		COR (95% CI)	*P* value	AOR (95% CI)	*P* value
Age (year), mean ± SD 44 (±18)		1.017 (1.000–1.035)	0.041	1.006 (0.980–1.032)	0.638
Educational status	No formal education	0.873 (0.248–3.065)	0.832	0.618 (0.115–3.305)	0.574
	Primary	0.267 (0.076–0 .933)	0.039	0.266 (0.053–1.338)	0.108
	Secondary	0.589 (0.397–0.173)	0.397	0.939 (0.188–4.676)	0.939
	Tertiary	1			
Hospitalization during the 3 months before the study period	No	1			
	yes	3.086 (1.472–6.466)	0.003	3.200 (0.927–11.047)	0.066
Previous diagnosis of infectious diseases	No	1			
	yes	2.003 (0.979–4.097)	0.057	1.639(.562–4.779)	0.366
Previous diagnosis of circulatory system diseases	No	1			
	yes	2.34 (1.080–5.066)	0.031	1.612 (0.507–5.126)	0.418
Diagnosis of blood and immune diseases during the study period	No	1			
	yes	1.837 (1.012–3.334)	0.046	3.925 (1.709–9.013)	**0.001**
Diagnosis of nervous system diseases during the study period	No	1			
	yes	2.951 (1.202–7.247)	0.018	1.649(.407–6.669)	0.483
CCI score		1.198 (1.033–1.389)	0.017	1.250 (0.961–1.624)	0.095
LOHS (days)		1.073 (1.028–1.119)	0.001	1.066 (1.009–1.125)	**0.021**
Previous medication prescription history	No	1			
	yes	2.075 (1.136–3.791)	0.018	1.054 (0.349–3.185)	0.925
Cardiovascular medicine prescription history	No	1			
	yes	2.201 (1.657–7.897)	0.001	2.040 (0.392–10.599)	0.396
Antibiotics prescription history	No	1			
	yes	1.675 (0.875–3.207)	0.119	1.894 (0.654–5.481)	0.239
Being on medication at admission	No	1			
	yes	2.689 (1.437–5.035)	0.002	0.891 (0.218–3.635)	0.873
Antibiotics prescription during the study period	No	1			
	yes	1.566 (0.818–2.998)	0.176	1.057 (0.449–2.483)	0.899
Cardiovascular medicines during the study period	No	1			
	yes	2.632 (1.199–5.776)	0.016	3.368 (1.137–9.979)	**0.028**
Antiepileptic prescription during the study period	No	1			
	yes	6.502 (2.083–20.291)	0.001	5.44 (0.962–30.745)	0.055
Analgesic agents’ prescription during the study period	No	1			
	yes	3.339 (0.931–11.976)	0.064	0.739 (0.110–4.951)	0.755
Gastrointestinal medicines prescription during the study period	No	1			
	yes	1.588 (0.863–2.923)	0.137	0.774 (0.335–1.786)	0.548
Number of medications prescribed during the hospital stay		1.409 (1.198–1.657)	< 0.001	1.310 (1.041–1.647)	**0.021**

*AOR*,Adjusted odds ratio, *CCI* Charlson’s comorbidity index, *COR* Crude odds ratio, *LOHS* Length of hospital stay

## Discussion

During the study period, patients, prescription orders, and laboratory results were meticulously monitored to pick up any incidences of ADEs. Overall, 64 ADEs were noted in 58 patients with an incidence rate of about 27 (95% CI, 21.03–32.30) per 100 admissions. This implies that ADEs pose a real burden on medical patients. Comparable incidences had been reported in multiple studies. Using a similar method, in a study by Takeshi M from Japan, 29.2 (95% CI, 27.7–30.7) incidence of ADEs were noted per 100 admissions [12]. Another prospective study by Ronald K involving Ugandan hospitalized patients confirms that the incidence rate of possible ADEs was 25 [95% CI: 22–29] per 100 admissions [14]. However, a study from Saudi Arabia reported that the incidence of ADEs was 8.5 [95% CI, 6.8–10.4] per 100 admissions [13]. This discrepancy might be explained by a variation in methods employed to detect ADEs. A study from Saudi Arabia relied mainly on information recorded in the medical records and heightened awareness by nurses only. Thus, some ADE incidences that were not documented in the medical record or otherwise reported might likely be missed.

In the present study, the relationship between the drug and events, as measured by the Naranjo algorithm showed 15.5% definite, 68.9% probable, and 15.5% possible, which is comparable with a prospective study from Morocco and the united kingdom (UK) where most of the events (67 and 66.5%, respectively) had probably related to the suspected medications [5, 6]. Using the same algorithm for causality assessment, a prospective study from India also identified about 71.9% ADEs were probable, and 26.1% were possible [44]. However, a cross-sectional study in Sweden reported fewer results of definite (1%) and probable (29%) ADEs [45]. These fewer results might be because of the variation in study design. A study from Sweden is a retrospective study, and thus missing some points which need subjective evaluation to know causality might happen.

In the study of ADEs, a key aspect is the possibility of prevention. In our study, more than half (59%) of ADEs identified were preventable. This implies that ensuring safe medication therapy is possible in the majority of patients admitted to medical wards. This finding is nearly similar to results from studies performed in Italy (69.4%) [6] and the United States (72%) [7]. Similarly, other prospective studies in the UK and Rabat showed that over half of the ADEs, 54 and 65%, respectively, were deemed possibly or definitely avoidable [5, 8]. However, the data from a multicenter observational study carried out in Netherland [36] showed lower preventability for ADEs (15.4%) compared to our study findings. This could be explained by the differences in the unit, as differences in the drugs used in the surgical inpatient setting could have contributed to these differences, and it might also be from differences in the health care systems.

According to the modified Hartwig ADEs Severity Assessment Scale, our study shows that most of the ADEs were mild (43.7%) or moderate (54.7%) in severity. This is in line with several other studies of hospital inpatients. A Prospective study by Davies E from the UK showed that approximately three-quarters of ADRs were scored at level 3 or below on the Hartwig scale [5]. Similarly, a study conducted in Ethiopia also agrees with our result, in which, 52.6% were moderate, and 37.1% were mild [15]. Contrary to the present study, in a multicenter, retrospective cohort study from Massachusetts, United States of America, about half of ADEs were rated as severe (serious in 49.4% and life-threatening in 11.7%) [46]. This inconsistency in severity might be due to the difference in the ward settings considered; all admission services were included except for the psychiatric and neonatal services in the later study. The higher mean age (52.5 years) of the patients in the later study could also relate to differences in results. Older patients take more medications and are more vulnerable to specific medication adverse effects than younger patients.

The most frequent system organs influenced by ADEs in our study were in line with other recent studies. In the present study, the gastrointestinal (27%) and central nervous systems (23%); followed by the endocrine and metabolic (11%), and cardiovascular systems (11%) were among the most frequently affected organ systems. This result is consistent with reports of a study conducted in four tertiary care public sector hospitals in Pakistan, in which the gastrointestinal tract accounted for one-third (33.3%) of organ systems affected by ADEs [47]. Similarly, a study from India showed that the gastrointestinal tract (51.7%) is the most commonly involved system [48]. Also in agreement with the current study, gastrointestinal (46%) and neurological (23%) disorders were the commonest system organ classes affected in Uganda [14]. Another prospective study in Rabat showed that metabolic disorders and cardiovascular (20.8% each) are system organ classes affected by ADEs [8].

Multivariate analysis showed that LOHS, use of cardiovascular medicines, diseases of the blood and immune, and the number of medications predicted the occurrence of ADEs in this study. As LOHS increases, the risk of occurrence of ADEs increased by 1.07 times (*p* = 0.019). Similarly, in a prospective cohort study from Japan, a significantly high length of hospitalizations (*p* < 0.01) was reported in patients who experienced ADE than in those who did not, with shorter stays for the latter group [49]. A Prospective analysis from the UK reported that the median length of stay for patient episodes that resulted in an ADEs was 20 days, compared to 8 days (*p* < 0.0001) for those episodes without ADEs [5]. Prospective observational studies in children and adults in Ethiopia revealed that patients with a prolonged length of stay were more associated with the occurrence of ADEs [15, 50]. This could be explained as patients with longer LOHS suffer from more severe conditions, multiple comorbid diseases, and had a higher dose of different drugs.

In our study, patients who use cardiovascular medicines were about four times more likely to experience ADEs (*p* = 0.014) than those without these medications. This is in line with a similar study conducted in the USA [46]. Other studies by Claudio B., and Rozenfeld S., also stated that cardiac therapy (9.6 and 28% respectively) is a therapeutic group that is frequently associated with ADEs [6, 15]. Thus, cautious prescription and frequent review of this class of medication is imperative.

The present study revealed that disease of the blood and immune is one of the significant predictors of ADE incidences. In agreement with this, a study conducted in Lima Peru found that anaemia, which is in the blood and immune diseases category, was independently associated with ADEs incidence (*p* = 0.001) [51]. Another study from Ethiopia by Merid also showed that patients who had anaemia were three times more likely to experience ADEs than patients who had no anaemia [52]. The more the medications that are prescribed, the more possibility of ADE incidences [53]. Likewise, in our study, the more medications that are prescribed for a patient, the greater the risk of ADEs (*p* = 0.027). Comparable to this, a prospective study by Nguyen T., in France confirms ADEs are significantly associated with the number of prescribed drugs (*P* < 0.001) [54]. Also, in a study from Brazil, the use of ten or more medications was associated with the occurrence of ADEs (*p*-value < 0.01) [55]. Our finding was also in agreement with studies conducted in Saudi Arabia and Uganda [14, 56]. Similarly, a study in the UK showed that the number of medicines taken was significantly higher than in those episodes not associated with an ADR (*p* < 0.001) [5]. This is because the prescribing of multiple drugs increases the risk of drug-drug interactions and the additive effects of multiple medications.

### Strength and limitations of the study

The strength of this study involves: 1) the prospective follow-up of the admitted patients allowed a more reliable recording of both the medication history and symptoms and the assessment of causality and using of standard scales given. It has helped to capture those ADE incidences which might have been missed with retrospective chart review study designs. 2) This study has employed a standard trigger tool besides the traditional way of identifying ADEs using self-report only. This increases the probability of detecting ADEs. This study is not without limitations. 1) Though statistically acceptable, the sample size in the present study is small, which could have decreased the power of the study. 2) The inclusion of a single academic hospital could also limit the generalizability of the study results. 3) To be certain of the ADEs causality, besides other scores, detection of some laboratory specimen concentrations of the medicine should be applied. But this is not done in our setting, which may affect the causality score. 4) Unfortunately, we employed Schumock and Thornton criteria on the bedside for spot ADE preventability assessment only and didn’t collect specific elements fulfilled under each of the three sections, namely definitely preventable, probably preventable, and non-preventable on the data collection tool during the study period. 5) There might be recall bias, especially in those patients who do not have a patient card.

## Conclusion

This study revealed ADEs incidences in about one-quarter of the participants. Longer hospital stays, blood and immune diseases, cardiovascular medicines, and multiple medication use had increased the likelihood of ADE occurrences. More than half (59%) of the ADEs were preventable. Based on findings, targeting hospitalized patients with an extended length of hospital stay, receiving cardiovascular medicines, multiple medication users, and proactive disease prevention, especially blood and immune diseases could help wean ADE incidences. Furthermore, the authors recommend a prospective and multicenter study addressing the limitations listed in this study.

## Supplementary Information

**Additional file 1.**

## Data Availability

Data generated and/or analyzed during this study are included in this article and its supplementary file [Additional file 1: ADE Dataset.csv]. In csv file all dates are mentioned in Ethiopian calendar. The dates are converted to Gregorian calendar in this article.

## Abbreviations

ADEs: Adverse Drug Events
AOR: Adjusted odds ratio
CCI: Charlson’s comorbidity index
COR: Crude odds ratio
ICD: International Classification of Diseases
LOHS: Length of hospital Stay
MONARCS: Modified Naranjo causality scale
SD: Standard deviation
WSUTRH: Wolaita Sodo university teaching referral hospital
